# Not All T Cell Synapses Are Built the Same Way

**DOI:** 10.1016/j.it.2019.09.009

**Published:** 2019-11

**Authors:** Sudha Kumari, Huw Colin-York, Darrell J. Irvine, Marco Fritzsche

**Affiliations:** 1 Koch Institute for Integrative Cancer Research, Massachusetts Institute of Technology, Cambridge, MA 02139, USA; 2 MRC Human Immunology Unit and Wolfson Imaging Centre Oxford, Weatherall Institute of Molecular Medicine, University of Oxford, Oxford OX3 9DS, UK; 3 Kennedy Institute for Rheumatology, University of Oxford, OX3 7LF Oxford, UK

## Abstract

T cells comprise functionally diverse subtypes. Although activated via a conserved scheme of antigen recognition by their T cell receptor, they elicit heterogeneous activation and effector responses. Such functional diversity has been appreciated in gene expression studies, functional assays, and disease models. Yet, our understanding of the principles underlying T cell subtype-specific activation and antigen recognition in the immunological synapse remains limited. This is primarily due to difficulties in primary T cell visualization at high spatiotemporal resolution and the adoption of tractable transformed T cell systems for cell biological experiments that may not correctly represent primary T cell constitutional diversity. Here, we discuss recent findings regarding the architectural and dynamic diversity of the immunological synapse and state-of-the-art methodologies that can be utilized to provide clues on how biological and biophysical differences in synaptic make-up could govern functional divergences in T cell subtypes.

An event common to all T cell subtypes is that their T cell receptor (TCR) recognizes a cognate peptide (p)MHC, leading to the establishment of a specialized cell–cell conjugate interface, termed the immunological synapse (IS), during an immune response [1]. In the limited T cell types studied to date, the dynamics and architecture of the IS are major determinants of antigen recognition and signaling; yet, the cellular principles that contribute to diverse IS patterns observed in distinct T cell subtypes remain largely unknown. However, this knowledge is required to understand how T cell subtypes developed functional specialization and to identify conserved principles of T cell activation that might be targeted to ideally enhance immune function.

While the underlying mechanistic details are unclear, evidence for distinctions in synaptic architecture and dynamics in T cell subtypes are compelling; both the nature of the antigen-presenting cell (APC), the surrounding microenvironment, and the T cell subtype are known to be important determinants of synaptic organization. Originally, a ‘bull’s eye’ IS pattern was described in CD4^+^ T cells using supported lipid bilayers or upon interaction with B cells 1, 2: central accumulation of the TCR was encircled by a ring of adhesion molecules forming the central super-activating molecular cluster and peripheral super-activating molecular cluster, respectively. A variation on the classical IS was observed when CD4^+^ T cells were activated using dendritic cells, resulting in a multifocal synapse [3]. These studies indicated that T cell subtypes exhibited diverse IS organization when recognizing different activation contexts (Figure 1A). Conversely, T cell subtypes can also form IS structures of remarkably different spatial and dynamic characteristics when using identical APCs. For instance, while CD4^+^ T cells can form a sedentary stable IS, CD8^+^ cytotoxic T cells (CTLs) can form a motile meta-stable IS, and regulatory T cells (Treg) appear to form the most stable IS of the three cellular subtypes when activated on reconstituted lipid bilayers, despite the fact that they all form bull’s eye IS structures under these experimental conditions 4, 5.

**Figure 1:**
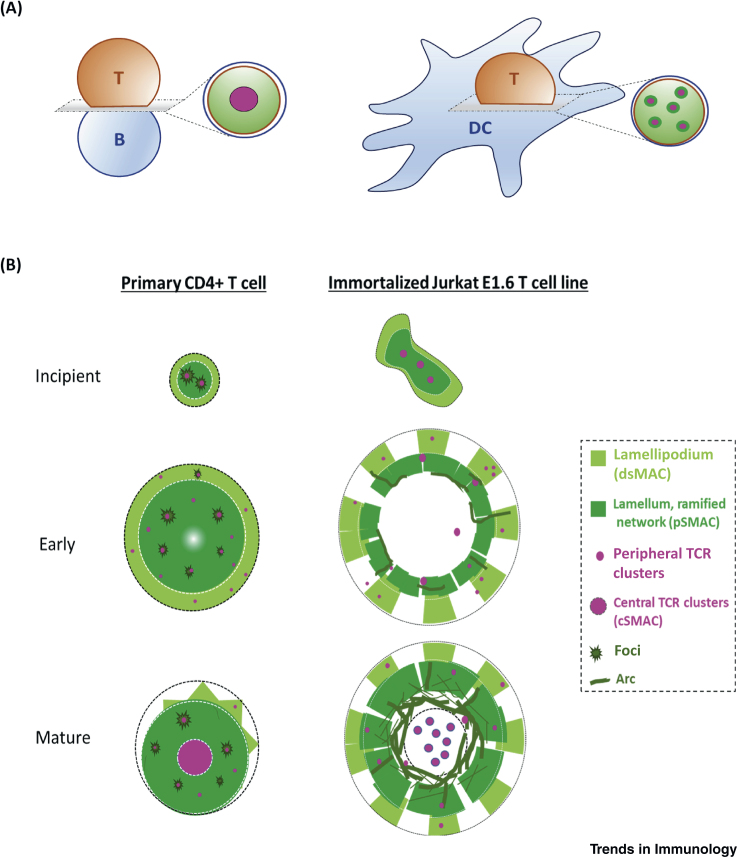
Schematics Illustrating Immunological Synapse Divergence in CD4^+^ T Cells. (A) Distinct synaptic pattern of CD4^+^ T cell–B cell (bull’s eye) versus T cell–DC (multifocal) synapse. (B) Evolution of T cell–SLB synapse of a typical primary CD4^+^ T cell or Jurkat CD4^+^ T cell line over time. The mature form of the synapse in primary T cell exhibits a prominent lamellum, foci, a ramified actin network, an ill-defined lamellipodium, and indistinct actin arcs. By contrast, Jurkat T cell synapses show a well-defined lamellum, lamellipodium, actin arcs and a ramified actin network [10]. Abbreviations: DC, dendritic cell; dSMAC, distal super-activating molecular cluster; pSMAC, peripheral super-activating molecular cluster; TCR, T cell receptor.

From a biophysical standpoint, the stability of the IS is accompanied and influenced by mechanical forces [6]; these are a physical consequence of spatial rearrangements at the IS. Indeed, the distribution, strength, and evolution of these forces likely depend on the architecture and dynamics of the IS structure in T cell subtypes [7]. Research over the past decade supports a major role for mechanical forces in T cell activation involving TCR-pMHC interactions, receptor triggering, signal initiation, and IS formation [8]. These processes often rely on mechanisms of mechanical feedback allowing T cells to scale and adjust their function to the activation context and microenvironment. Differences in the interaction of T cell subtypes with APCs are likely to involve the actin cytoskeleton; a primary determinant of the biophysics and mechanics of the IS. We speculate that the actin cytoskeleton can mediate a force balance across the synaptic interface to control the dimensions and lifetimes of various subsynaptic zones; these in turn, may alter different steps of T cell activation 8, 9.

Currently, we only have a limited understanding of how cytoskeletal actin organization influences distinct synaptic patterning at the IS. This is largely due to limited transfection/expression of genetic reporters of actin architecture and dynamics in primary lymphocytes, the small size of synapses, and the active cellular interface that precludes visualization at high spatiotemporal resolution. To overcome some of these limitations, Jurkat T cells have been often adopted as a cellular model of choice. Not surprisingly, even under identical activation and ligand density conditions, Jurkat T cells and primary CD4^+^ T cells have revealed distinct synaptic patterns [10]. For example, the filamentous actin (F-actin) architecture and dynamics of Jurkat T cells have been found to differ considerably from their primary CD4^+^ T cell counterparts (Figure 1B). Specifically, primary CD4^+^ T cells have shown four distinct actin organization patterns at the IS; the prominent lamellum, the actin foci [11], the ramified actin network 12, 13, and the somewhat indistinct lamellipodium. By contrast, Jurkat T cells have shown a prominent lamellipodium, a ramified actin network, and a smaller lamellum, where actin foci are practically absent [10]. In addition, actin arcs, a prominent feature of the Jurkat and CD8^+^ T cell F-actin network [14], appear to be far less conspicuous in primary CD4^+^ T cells [10]. Hence, while these architectural differences suggest that actin network dynamics, mechanics, and rheology are likely to be different in these cells, they might also influence how T cells employ mechanical feedback during antigen recognition [9]. These differences also highlight the fact that care should be practiced in generalizing the cellular mechanisms underlying the diversity of IS patterns and motility behaviors for specific T cell subtypes and across species.

To gain a deeper understanding into how individual T cell synapses are assembled, the biomedical community should aim to further strengthen its interdisciplinary efforts integrating the latest technological developments: new advances in genetic tools, computational methods, and super-resolution microscopy techniques are beginning to enable the interrogation of diverse primary T cell subtype synapses from the bottom-up, interlinking findings from the molecular, cellular, and immune system levels [15]. Visualization and quantification of cytoskeletal actin dynamics underlying IS patterns and their kinetic progression has evolved to a remarkable degree in terms of spatiotemporal resolution. Minimally invasive and fast acquisition imaging, such as by lattice light sheet microscopy, live-cell super-resolution microscopy, and force probing techniques, is currently transforming how we observe cellular and protein arrangements during T cell APC interactions.

In our view, the systematic application of the latest microscopy and mechanobiology technologies can provide a powerful toolbox to unravel the complex principles underlying the organizational and functional diversity of T cell subtypes; it is evident that T cell subtypes can employ different ways of building an IS. Future research should focus on how this diversity is achieved and which biochemical and mechanical characteristics dictate it. Furthermore, work is needed to understand how such functional specialization benefits T cells and whether the macro-scale architecture of the synapse can influence the direct cytoskeletal and/or mechanical feedback applied to membrane receptors; this may alter their dwell time, affinity thresholds, or a combination of the two [15]. Additionally, synaptic organization could also impact the lifetime of the IS, the latter being crucial for T cell effector functions such as delivery of cytotoxic granules in CD8^+^ T cells or inhibition of APCs by Treg cells. Studying actin cytoskeletal organization and dynamics in different primary T cells populations will be a good starting point to pursue answers for some of these questions and help unravel ways in which the cytoskeleton has adapted to serve subtype-specific synaptic functions. Studies over the years have established actin dynamics as a crucial mediator of TCR activation and mechanical forces at the IS. Thus, recent advances in microscopy can enable a better assessment of the nature and localization of mechanical forces exerted at divergent synapses in fine detail, at the receptor level or intracellularly, suggesting whether tensile, compressive, protrusive, or frictional forces are dominant. Furthermore, although pending further validation, preliminary data suggest that certain immunodeficiency diseases (such as Wiskott-Aldrich syndrome and Dedicator of cytokinesis 8 deficiency) have been mapped to the lesions in actin regulatory proteins [16]. Understanding the cytoskeleton and associated mechanical forces in T cells in the context of these diseases should help bridge our knowledge of the molecules that execute divergent T cell synaptic patterns; such patterns could be potentially relevant in the physiopathology of such diseases.
